# A Computational Model of Watermark Algorithmic Robustness Capable of Resisting Image Cropping for Remote Sensing Images

**DOI:** 10.3390/s18072096

**Published:** 2018-06-29

**Authors:** Deyu Tong, Na Ren, Wenzhong Shi, Changqing Zhu

**Affiliations:** 1 Key Laboratory of Virtual Geographic Environment, Nanjing Normal University, Ministry of Education, Nanjing 210023, China; 141301013@stu.njnu.edu.cn (D.T.); 09322@njnu.edu.cn (C.Z.); 2 State Key Laboratory Cultivation Base of Geographical Environment Evolution, Nanjing 210023, China; 3 Jiangsu Center for Collaborative Innovation in Geographical Information Resource Development and Application, Nanjing 210023, China; 4 Department of Land Surveying and Geo-Informatics, The Hong Kong Polytechnic University, Kowloon, Hong Kong 999077, China; lswzshi@polyu.edu.hk

**Keywords:** digital watermarking, robustness, computation model, image cropping

## Abstract

Various watermarking algorithms have been studied to better enable the copyright protection of remote sensing images. The robustness of such algorithms against image cropping attacks has subsequently been verified mainly by various experiments. However, to date, the experimental results are subject to the differences in experimental factors and computational resource costs. Hence, the study presented in this paper proposes a robustness computation model of watermarking remote sensing images in terms of the image cropping attack. The robustness computation model consists of three parts: analysis principles, an evaluation index, and a computation method. The robustness analysis principles are provided based on the salient features of watermarking remote sensing images and attacking properties. A probability-based evaluation index is then defined to more comprehensively measure the robustness of different algorithms. The computation method developed in this study is based on permutations and the inclusion-exclusion principle to theoretically calculate robustness. The experiments conducted to verify the effectiveness of the computation model, revealed true closeness between both the calculated and experimental results. Finally, the relationships between the robustness and the different parameters used in the watermarking algorithms are investigated by using the proposed computation model.

## 1. Introduction

Currently, remote sensing images are widely used in scientific research and various industries, including agriculture, vegetation monitoring, ecology and so on [1,2,3]. However, security issues inhibit the convenience of remote sensing image sharing and transmission [4], especially in the big data era [5]. Robust watermarking technology has become a promising solution, which could be a viable form of protection regarding issues such as user tracking, forgery prevention and many other illegal uses [6,7,8,9,10,11]. The major property of these watermarking algorithms, named robustness, represents the resistance towards different types of attack [12,13,14]. As the image cropping operation is the common processing method towards remote sensing images, the watermark algorithmic robustness against image cropping attack is evaluated in almost every watermarking algorithm study [10,11,15,16]. In fact, this type of robustness determines whether the copyright can be successfully identified after cropping a certain amount of remote sensing image.

It is common to verify the watermarking algorithmic robustness against image cropping attacks by experimentation, both for common images and remote sensing images. From the perspective of computational complexity, the experimental methods can be divided into three types: robustness verification, algorithm comparisons, and criteria systems. The robustness verification experiments are mainly conducted after the watermarking algorithms have been proposed by performing some attacks [8,10,11,17,18,19,20,21,22,23]. Normalized correlation (*NC*) [10,17,23] and bit error rate (*BER*) [17,24] have been used as the evaluation indexes, which are calculated by comparing the extracted copyright information with the original. Thus the watermarking algorithmic robustness is determined conveniently and directly in this method. To demonstrate the robustness in a more reliably persuasive way, algorithm comparisons verify the robustness result of those proposed watermarking algorithms and others [8,18,21,22,23]. In addition, the robustness criteria systems and platforms, which defined a variety of sample data, attacking types (including image cropping attack), attacking strength and other parameters, have been studied and published. These criteria systems, including “Stirmark” [25,26], Watermark Evaluation Testbed (WET) [27] and Information Hiding and its Criteria for Evaluation (IHC) [28], strive to extend the range of experimental parameters and conditions to more accurately estimate the robustness of the watermarking algorithms. For example, resistance to image cropping attack is considered as the most basic and low-level type of robustness in the Stirmark Benchmark evaluation system [26], and image databases with strategies of repeated tests have been adopted [29].

Unfortunately, as indicated above, it is obvious that the experimental method of evaluating robustness consumes many computational resources and much time. With the different parameters and experimental conditions, the computational resources and time used to evaluate the robustness will thus, increase further, especially for the remote sensing images in the big data era. Further, the experiments cannot cover all kinds of conditions as the parameters related to sample data, attacking type, attacking strength and so on. Thus, the robustness of the experimental results can only be used to approximate robustness ability and cannot, in theory, deduce the robustness index value.

The significance to theoretically analyze and calculate the robustness of watermarking algorithms against image cropping attack is believed to be important and therefore necessary for further study. Compared to research on watermark embedding and extraction strategies, not much attention has been paid to robustness analysis and its calculation methods. As regards the watermarking model, Adelsbach gave the definitions of the watermarking schemes and provided an abstract model of watermark embedding, extraction, and robustness [30]. Regarding the embedding strategy, the quantization modulation has theoretically proved its superiority in robustness [31]. In terms of watermark capacity analysis, Sun provided the closed formula for calculating the watermark capacities of the private Laplacian watermarking system [32]. According to the robustness analysis and computation, Hu proposed the quantitative relationship between robustness and destructed watermark units [33].

It should be noted that there are still two drawbacks to realize the computation of robustness under image cropping attack: (1) Quantitative models regarding robustness and image cropping attacks have not been comprehensively established. Some analyses of watermarking algorithms, in fact, focus on the watermarking model [30], embedding strategy [31] or watermarking capacity [32], rather than the robustness. The work accomplished by Hu simplified the attack parameter as the ruined watermark bits count [33], which bypassed the analysis of attacking properties and attacking strength; (2) The evaluation index of robustness is not universal, particularly in terms of comparisons. The commonly used indexes, including *NC* or *BER*, only represent the robustness in one single experiment. In this sense, these indexes vary according to the experimental data and conditions. Consequently, each robustness result from the research cannot be literally and directly compared.

Unlike the intentions of previous works, this study proposes a computation model to directly analyze and calculate the watermarking algorithmic robustness under image cropping attack. The computation model involves analysis principles, a robustness evaluation index and a computation method. The analysis principles are concluded from well-designed watermarking algorithms and a robustness index is defined based on probability. According to the proposed principles and index, a computation method is presented, based on the approaches of combinatorial mathematics and probability theory. In addition, the influences of different factors, related to the watermarking algorithm on robustness are further explored with the object of promoting the design of watermarking algorithms.

The rest of this paper is organized as follows: Section 2 introduces the methodology of robustness computation model. In Section 3 and Section 4, the experimental results and discussions are given respectively. Finally, conclusions are given in Section 5.

## 2. Methodology

**Figure 1:**
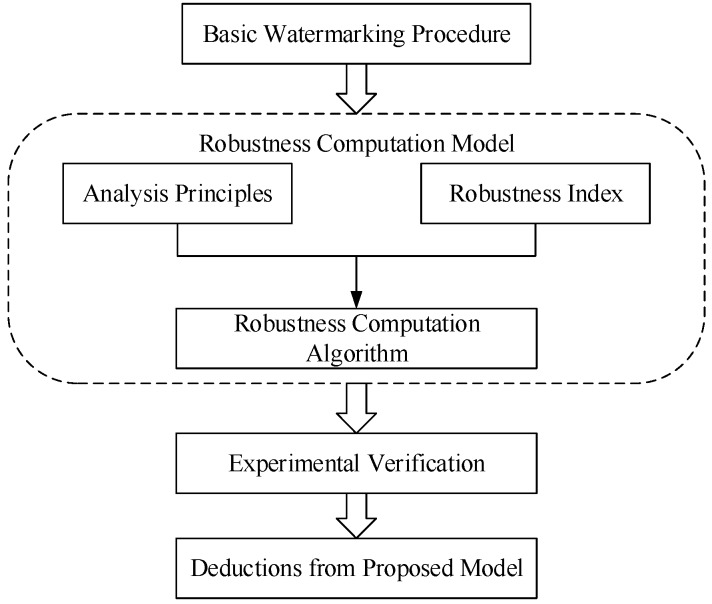
Research diagram to show the robustness computation model.

The research diagram of robustness computation model is illustrated in Figure 1.

The basic watermarking procedure is given previously. Then the computation model, consisting of analysis principles, robustness index and robustness computation method, will be proposed and clarified. Finally, experiments and deductions are conducted to verify the proposed model and promote the design of watermarking algorithms for remote sensing images.

### 2.1. Basic Watermarking Procedure

**Figure 2:**
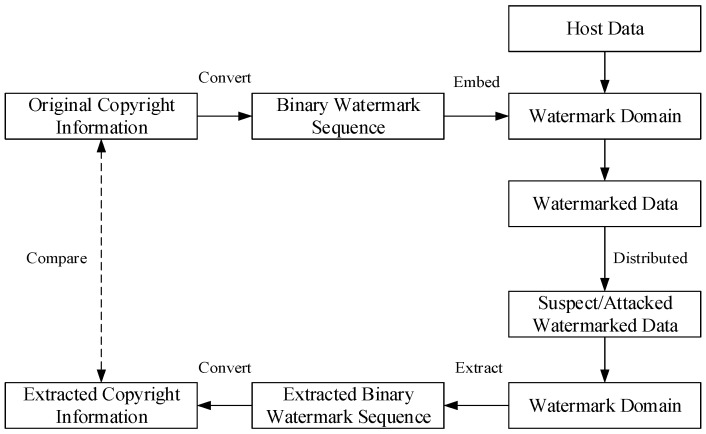
Watermarking procedure.

The basic watermarking procedure and fundamental knowledge are presented here. A classical watermarking procedure is shown in Figure 2.

In the watermarking procedure, the original watermark information is converted into binary watermark sequence, then embedded into the watermark domain, which is transformed or generated from the host data. After watermarked data has been delivered or distributed, the copyright information is extracted by the inverse method and compared to the original one. Thus, the copyright can be identified clearly as long as the watermarking algorithm is sufficiently robust.

It has to be pointed that the copyright information includes text, voice, image and so on, and it is converted into binary sequence before the process of embedding. In addition, the comparison of copyright information is equal to the comparison of binary sequence as long as the conversion is reversible. Thus binary watermark sequence is regarded as the form of what watermarking algorithm embed and extracted in this paper. Watermark synchronization and robustness evaluation index, which are closely related to the issue of robustness computation for image cropping attacks, are discussed as follows.

#### 2.1.1. Watermark Synchronization

The watermark synchronization is the vital factor related to robustness when a watermarking algorithm suffers the geometric type of attack such as image cropping, image rotation and so on. In fact, sometimes the watermarked values can remain correct but the error of synchronization will lead to the failure of watermark extraction. Specifically, consider the watermark sequence *WM* being represented as:

(1) WM=wm[i](wm[i]∈{0,1}  i=1,2,…,L),

where *L* is the length of *WM*. Under geometric attack, embedding *wm*[*i*] and directly extracting it could lead to the loss of *i*. So *i* is an important information part in both processes of watermark embedding and extraction. Therefore, the connections between *i* and *wm*[*i*] represent the watermark synchronization. Commonly, a stable watermark synchronization definitely ensures the watermarking algorithm robustness against image cropping attack. So far this mechanism has been implemented in different ways, such as geometric reference points [6], auxiliary location information [10,34], mapping method [35], etc.

#### 2.1.2. Robustness Evaluation Index

Evaluating a watermarking algorithm is commonly performed from several perspectives, e.g., peak signal-to-noise ratio (*PSNR*) is used to measure the degradation of image fidelity [13,36], false negative probability (missed detection) or false positive probability (false alarms) are used to evaluate the authority and authentication of watermarking algorithms [15,37]. Regarding robustness, it is common to use *NC* [10,17,23] or *BER* [17,24] to compare the similarity between the extracted watermark sequence with the embedded one in a quantitative method. For further evaluation, taking *BER* as an example, assume *wm* is the original watermark sequence and $wm^{\prime \prime }$ is the extracted, *BER* is calculated as:

(2) $$BER=\frac{1}{L}\sum_{i=1}^{L}NOR(wm^{\prime \prime }[i],wm[i]),$$

where *NOR* is the operation of exclusive *OR*. BER=0 means there is no error in the extracted watermark. Commonly the threshold of *BER* is set previously and empirically in robustness experiments.

### 2.2. The Principles of Robustness Computation

Based on the basic watermarking procedure, the principles are deduced through the analysis of watermarking algorithm and the attack behavior. Hence, these principles are divided into principles of watermarking algorithm and principles of image cropping attack. It can be seen that the principles are universal and do not have many constraints as they are proposed based on few essentially natural assumptions.

#### 2.2.1. Principles of Watermarking Algorithm

• Principle 1: The watermark is repeatedly embedded into data.

The volume of the remote sensing image is commonly much larger than the length of binary watermark sequence, thus the watermark is usually embedded more than once into data for robustness enhancement. It is well known that this principle has been applied in current watermarking algorithms [8,38,39,40,41]. In fact, the repeat embedding strategy can be regarded as the spread spectrum [15], in that the former is a point of view in the spatial domain and the latter a strategy in the transformation domain. Considering that image cropping is conducted mainly in the spatial domain, thus the similar principle can be adequately used to express the strategy in the same domain for convenience.

**Figure 3:**
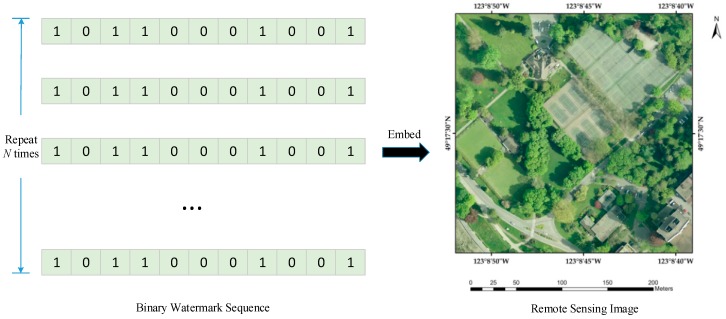
Illustration of Principle 1.

Specifically, repetition is represented by *N*, which means the watermark has been embedded *N* times in the host image or that watermark information has been spread *N* times in the transformation domain. To simplify the analysis principle and robustness computation model, *N* is constrained as positive integers. Figure 3 illustrates the concept of Principle 1.

• Principle 2: The watermark is distributed uniformly.

Because of the large size of the remote sensing image together with Principle 1, the watermark is equally distributed throughout the image. If the watermark energy is concentrated in a small part of the spatial domain or the narrow band in the transformation domain, the image fidelity, both in this area, together with the watermark robustness in other parts, will greatly degrade. Hence, Principle 2, which have been also adopted widely [8,10,15,41,42], represents the tradeoff between watermark imperceptibility and robustness.

**Figure 4:**
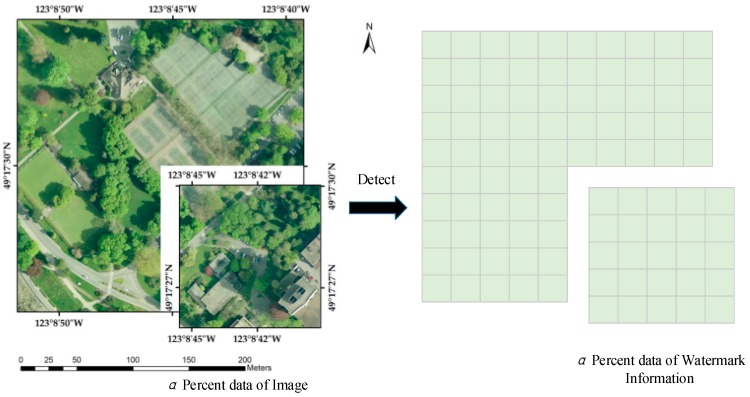
Illustration of Principle 2.

Quantitatively, if a watermark of *L* bits length is repeatedly embedded into the remote sensing image, for *N* times; in such a situation, Principle 2 suggests the watermark extraction will extract *a* × *L* × *N* bits of the watermark sequence from *a* percentage amount of the remote sensing image. This is made apparent in Figure 4. It should be pointed out, however, that it is not consistent for the extracted watermark bits in Figure 4 to be exactly situated, in the lower right-hand corner of the watermark information, as can be seen in Figure 3. As noted in Principle 2 the watermark information is not necessarily embedded sequentially. In Figure 4, the extracted watermark bits are regarded as randomly chosen from the whole watermark bits.

#### 2.2.2. Principles of Image Cropping Attack

**Figure 5:**
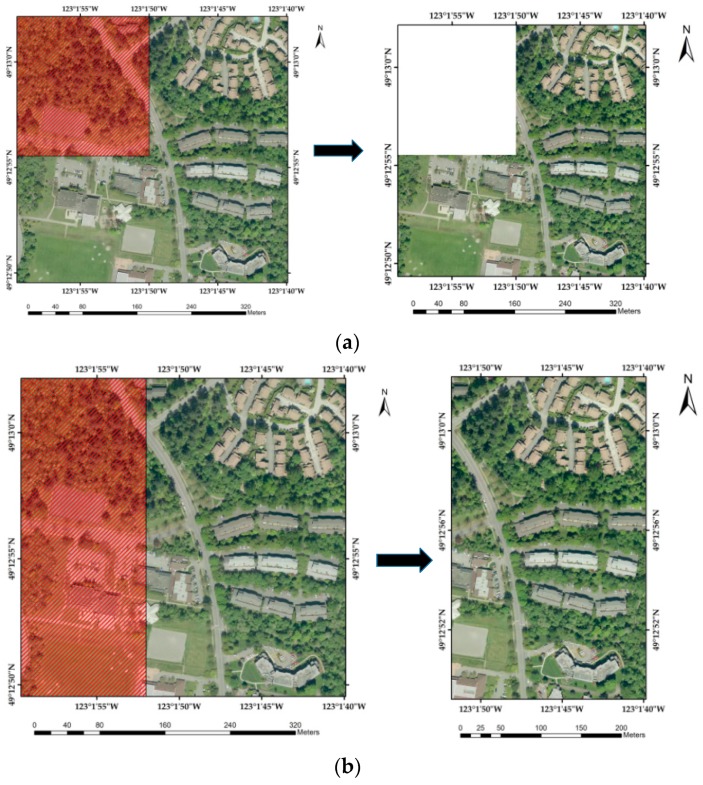

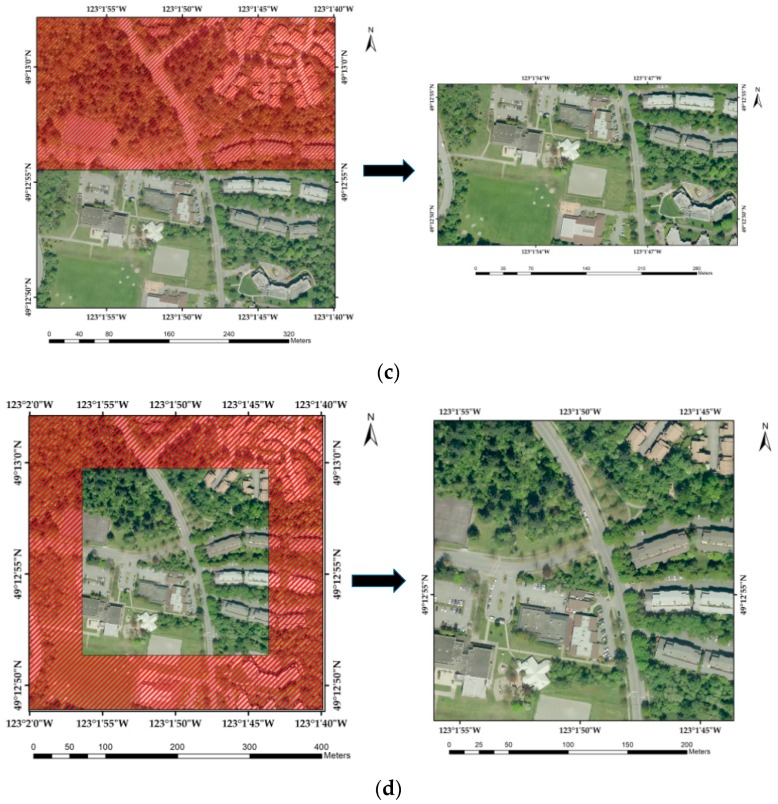
Examples of image cropping attack with different attack strengths: (**a**) Attack strength of 0.25; (**b**) Attack strength of 0.4; (**c**) Attack strength of 0.5; (**d**) Attack strength of 0.6.

Image cropping can be seen as a type of geometric attack. Part of the watermarked image is deleted randomly and watermark extraction is conducted in the remaining part. The attacking strength is defined as the percentage of the cropped data. Some examples of different attacking strength are shown in Figure 5.

Combine the attack behavior of image cropping with Principle 1 and Principle 2, the analysis principles of attack are deduced as follows:

• Principle 3: The watermark and its synchronization in the remaining part remain correct.

The watermark information contained in the cropped part, obviously, cannot be further retrieved. According to Principle 2, the uniformity guarantees the effectiveness of Principle 3 for both watermarking algorithms based on the spatial domain and those based on the transformation domain. After image cropping, the watermark synchronization mechanism becomes the key to guarantee the robustness. It appears obvious that a well-designed watermarking algorithm will reduce the attack influence on the remaining parts to a minimal level. Because watermark synchronization has different types of implementations [6,10,34,35], Principle 3 represents the ideal situation that all the watermark containing in the remaining part could be successfully extracted.

• Principle 4: The cropped part of image is chosen randomly.

**Figure 6:**
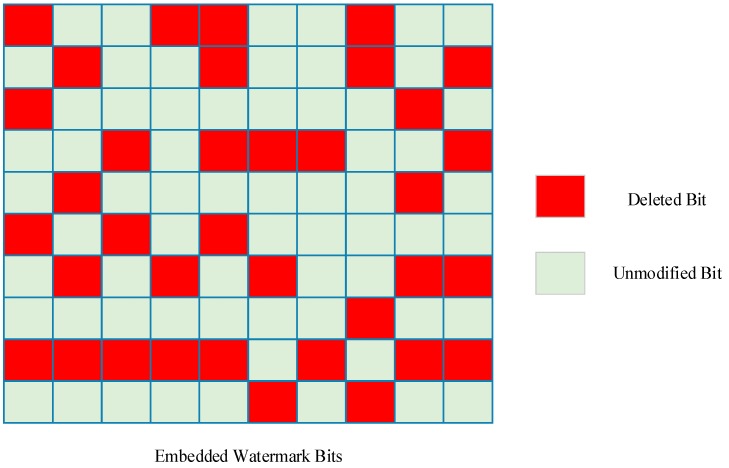
An example demonstrating Principle 4.

As shown in Figure 5, the cropped part of the image can be anywhere, even under the same attack strength. Because the cropped part is not fixed to a specific area for each attack, hence it is reasonable to assume that the cropped part is randomly chosen. In addition, according to the uniformity distribution in Principle 2, cropping image randomly equals a random deletion of the watermark information. An example is shown in Figure 6 to illustrate the influence of image cropping attack on watermark.

In Figure 6, the red blocks represent the deleted watermark bits, and the green blocks represent the correct. The deleted parts, the watermark information in cropped part of image, are distributed randomly in theory. According to Principle 3, the remaining parts of the watermark, which is denoted by green blocks, can then be correctly extracted.

### 2.3. Probability-Based Robustness Index

The second part of the computation model is the probability-based robustness index. As the common evaluation index, *BER* indicates the watermarking algorithmic robustness in a single experiment, the calculation method refers to Equation (2). It cannot be used, however, to directly compare the *NC* of different watermarking algorithms, due to the different experimental parameters and conditions. To more conveniently and comprehensively measure robustness, a quantitative index *R* is proposed from the perspective of probability. Here *R* is defined as the probability of successfully and correctly extracting the watermark information after attack according to the given threshold value BER*. As BER* is usually a constant value in the system of robustness evaluation, it is often omitted and index *R* is then expressed as:

(3) R(AT,S,L,N).

In Equation (3), *AT* represents the attack type, and in this paper denotes image cropping. *S* is the attacking strength indicating that cropping *S* percentage of data. *L* is the watermark information length and *N* represents the repeat times of the embedded watermark.

From the definition of index *R*, the superiorities of the proposed index, compared to traditionally used ones are explained as follows. First, robustness is evaluated by probability, thus the more times experiments are repeated under the same parameters of attack, the greater the likelihood that the evaluated value *R* will approximate to the theoretical value. Then, *R* has no relationship to the sample data. Hence this index has universal usage and contributes to the comparison of the robustness of different algorithms conveniently. In addition, providing the attacking strength is well defined, the proposed index is not limited to the image cropping discussed in this paper. Hence, this quantitative index is applicable to all kinds of attack. Accordingly, the universal index R can be used to evaluate robustness more scientifically and comprehensively.

### 2.4. Robustness Computation Method

Based on the principles and the evaluation index proposed above, the method to compute the watermarking algorithmic robustness is studied here. The main theory for solving this problem is the enumeration of the watermark distribution after image cropping attack, based on combinatorial mathematics. By dividing all the enumerations (denoted as *E_total_*) by the number of successful situations, from which watermarks have been correctly extracted (denoted as *E_suc_*), the extraction probability *R* is calculated directly as:

(4) $$R=\frac{E_{suc}}{E_{total}}.$$

The question is then becomes how to calculate *E_total_* and *E_suc_*. Obviously, the calculation of *E_total_* is easy to conduct according to the permutations, which will be given in the following section. However, the difficulty of this problem is to calculate *E_suc_*. From the deduction above, *E_suc_* can be acquired by enumerating the situations under image cropping attack with the watermark can still be extracted. But as far as we are concerned, counting the situations directly is easy to confuse some situations and complicated to give the computation formula correctly. In order to solve the problem, the auxiliary function has been adopted to calculate *E_suc_*.

#### 2.4.1. Introduction of the Auxiliary Function

Before the introduction and definition of the auxiliary function, the parameters in the computation are explained as follows. The evaluation index is expressed as *R*(*Cropping*, *S*, *L*, *N*). Here *S* represents the cropped data percentage and the threshold value of *BER* is set to *r*. According to Principle 1, the total embedded watermark bits are L×N. From Principle 3 and Principle 4, the process of cropping images is randomly deleting certain number bits of watermark information. Based on principle 2, the operation of cropping *S* percentage of remote sensing image is equal to deleting *S* percentage of embedded watermark bits. To simplify the computation, the number of cropped watermark bits, denoted as *D*, is calculated as the expected value, which is D=L×N×S. According to the *BER* calculation method and watermark synchronization, the watermark bit *wm*[*i*] will be properly extracted if any embedded watermark bits corresponding to *i* remain correct.

**Figure 7:**
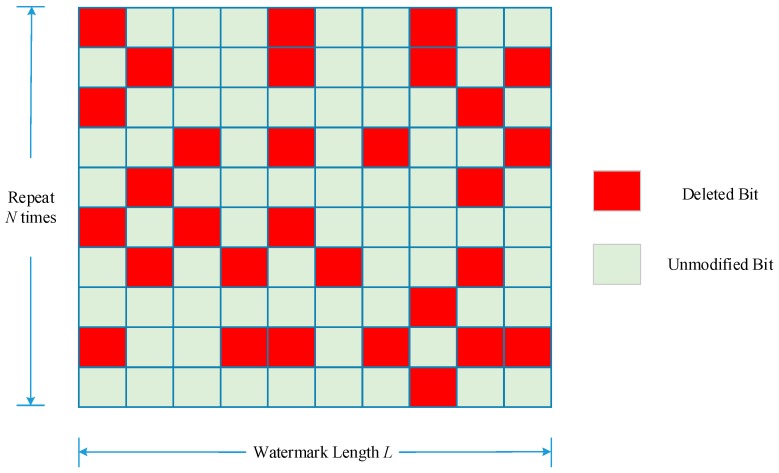
An example belongs to enumerations of function *P*.

**Figure 8:**
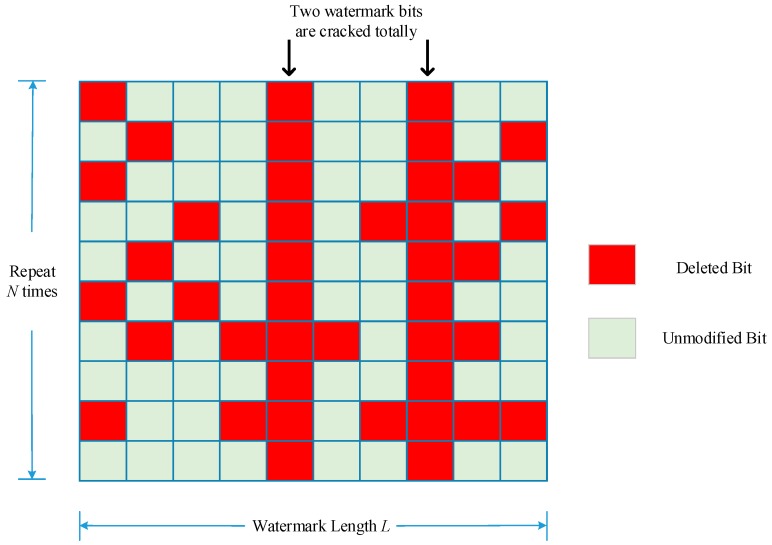
An example does not belong to enumerations of function *P*.

The auxiliary function *P*(*L*, *N*, *D*) is then introduced as the count of all enumerations of any watermark bit is not ruined totally when deleting *D* watermark bits from watermark information length *L* embedded over *N* times. Figure 7 illustrates an example belonging to the auxiliary function *P*, and Figure 8 shows an example not belonging to it as two watermark bits are totally cracked.

In Figure 7, all the blocks represent extracted watermark bits, where the green one means that the remaining watermark bit has not changed after the attack. The red one represents the cropped bits. In this case, 30 bits have been cropped but watermark information can still be extracted correctly providing any column in the matrix has not been cropped totally. However, the situation in Figure 8 does not belong to function *P* as two watermark bits are both deleted, the watermark information extracted from this situation will lose the two bits permanently.

#### 2.4.2. Computation of the Auxiliary Function

From the definition of auxiliary function *P*, it is obvious that function *P* cannot be calculated directly according to the accumulated counts when ruined watermark bits have increased from 1 to *L*. This is because the circumstances have intersections. To count the permutations *P(L, N, D)* without repetition and omission, the inclusion-exclusion principle of combinatorial mathematics is adopted. The inclusion-exclusion principle is expressed as follows. If there are sets $A_{1},A_{2},\ldots ,A_{n}$, the count of whole elements is calculated as:

(5) $$\begin{array}{l} \left|\overset{n}{\underset{i=1}{\cup }}A_{i}\right|=\sum_{i=1}^{n}\left|A_{i}\right|-\sum_{1\leq i<j\leq n}\left|A_{i}\cap A_{j}\right|+\sum_{1\leq i<j<k\leq n}\left|A_{i}\cap A_{j}\cap A_{k}\right|-\ldots \\ \;+{\left(-1\right)}^{n-1}\left|A_{1}\cap A_{2}\cap \ldots \cap A_{n}\right|, \end{array}$$

where the symbol | | represents the counts or cardinalities of the sets. By using the principle in auxiliary function *P*, the sets containing *i*-th watermark bit which has been ruined are denoted as $P_{i}(1\leq i\leq L)$, then the function *P* is:

(6) $$P(L,N,D)=C_{L\times N}^{D}-\left|\overset{n}{\underset{i=1}{\cup }}P_{i}\right|,$$

$C_{L\times N}^{D}$ represents the count of all the possible permutations when deleting *D* watermark bits from L×N watermark bits. So $\left|\overset{n}{\underset{i=1}{\cup }}P_{i}\right|$ is calculated according to Equation (5). Specifically, the function *P* (*L*, *N*, *D*) is calculated as:

(7) $$P(L,N,D)=C_{L\times N}^{D}-\sum_{i=1}^{M}{\left(-1\right)}^{i+1}C_{L}^{i}\times C_{(L-i)\times N}^{D-i\times N},$$

with $M=\left\lfloor \frac{D}{N}\right\rfloor$, the operation ⌊ ⌋ rounds the value down to an integer.

#### 2.4.3. Computation of Robustness Index

After the function *P* has been introduced, the robustness index *R*(*Cropping*, *S*, *L*, *N*) can be calculated in the theory. According to Equation (4), the formula of *E_total_* is given as:

(8) $$E_{total}=C_{L\times N}^{S\times L\times N}.$$

*E_suc_* can then be calculated as the enumerations where ruined watermark bits are smaller than the threshold value *r* with the help of auxiliary function *P*. The number of minimum correct watermark bits (denoted as *X*) is calculated by:

(9) X=⌈L×(1−r)⌉.

The operation ⌈ ⌉ rounds the number up to an integer. By changing the count of correct watermark bits from *X* to *L*, the robustness index *R*(*Cropping*, *S*, *L*, *N*) is calculated based on Equation (7):

(10) $$R(Cropping,S,L,N)=\sum_{i=X}^{L}C_{L}^{i}\times P(i,N,S\times L\times N-N\times (L-i))/C_{L\times N}^{S\times L\times N}.$$

Hence, the robustness index can be acquired based on the proposed formulae in numerical calculations.

## 3. Experimental Results and Analysis

Two types of experiments have been conducted. The first experiments aim to verify the effectiveness of the proposed computation model. The results calculated by that model are compared with the algorithm results. The second experiments are the deductions of that model. The deductions demonstrate the relationships of the robustness, watermark information length and watermark repeat times. All the experiments are conducted on MATLAB 2016a and ArcGIS 10.2. In addition, Multiprecision Computing Toolbox are used to guarantee the numerical accuracy in robustness computation. The MATLAB code of the proposed model is listed in Appendix A.

### 3.1. Materials

**Figure 9:**
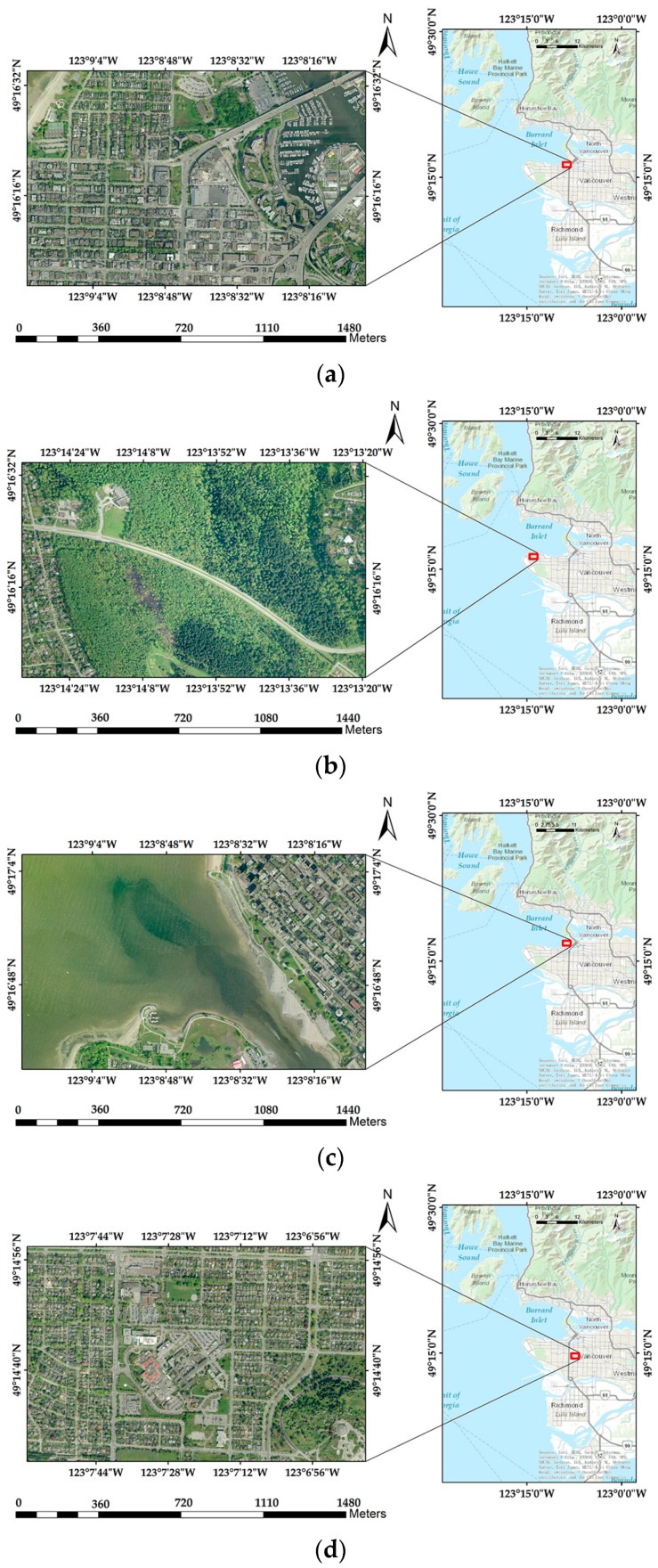
Parts of experimental data: (**a**–**d**) are remote sensing images of Vancouver.

**Figure 10:**
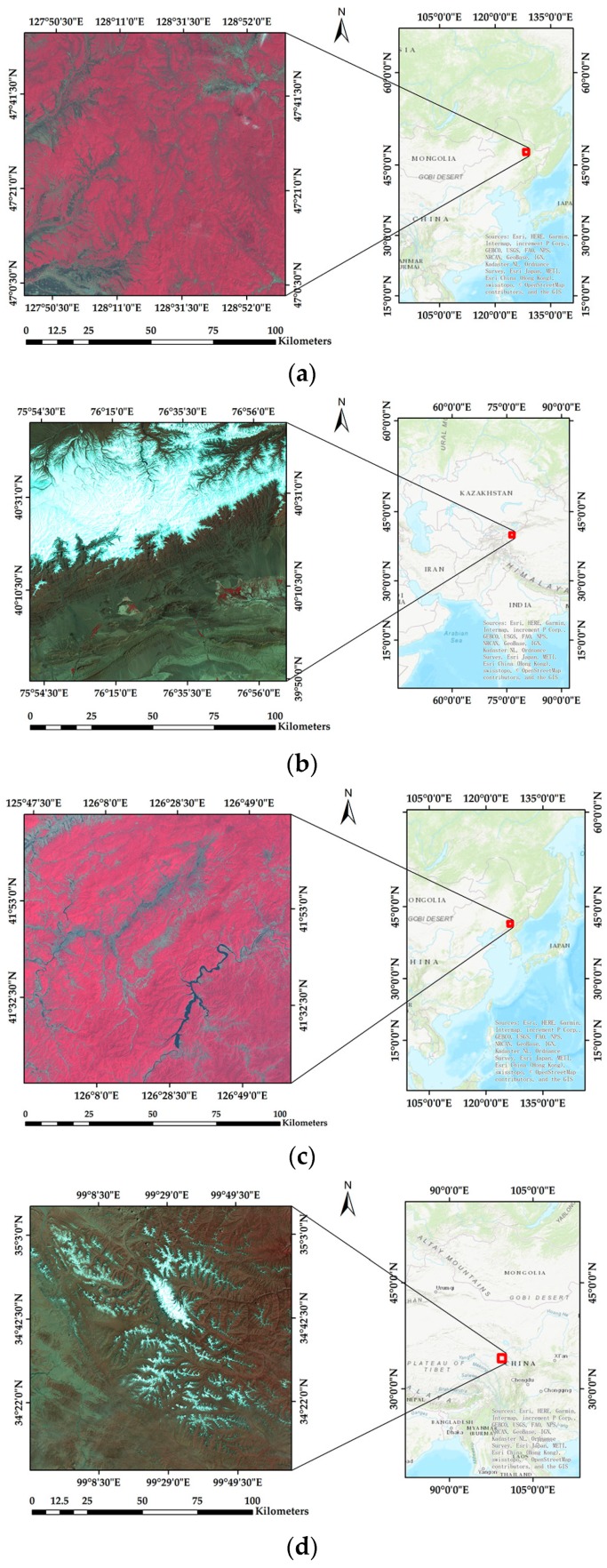
Parts of experimental data: (**a**–**d**) are remote sensing images of Landsat7.

In the first experiment, the evaluation indices are calculated based on the proposed theoretical model and watermarking algorithms respectively. The experimental data contains 20 remote sensing images of Vancouver and 20 remote sensing images from Landsat7 with sensors of Enhanced Thematic Mapper Plus (ETM+) in ScanLinesCorrector (SLC)–off mode. The images of Vancouver are the open datasets downloaded from the City of Vancouver website (available at http://data.vancouver.ca/datacatalogue/2011facetsGridECW.htm). Their resolution is 16,000 × 10,000 with three bands and the size is 457 MB in .tiff format. The images of Landsat7 are provided by Geospatial Data Cloud site, Computer Network Information Center, Chinese Academy of Sciences (http://www.gscloud.cn). Their resolution is 4096 × 4096 with nine bands and the size is 144 MB in .tiff format. Parts of the experimental data are shown in Figure 9 and Figure 10 with the overview map acquired from World Topographic Map of ArcGIS Online data.

### 3.2. Verification of Proposed Model

Three types of watermarking algorithms have been chosen because their embedding methods are nearly consistent with or easily adapted to satisfy the proposed watermarking principles. The first watermarking algorithm denoted as Algorithm A is based on scale-invariant feature transform, where embedding methods can be referred to [11]. Algorithm B is based on discrete cosine transformation, referred to [8] and Algorithm C is based on statistic index, referred to [10]. In addition, repeat embedding strategy and watermark synchronizations have been adopted in the watermarking algorithms in accordance with the proposed principles. The verification is conducted with three sets of experimental parameters and pseudo-random binary sequence are used as the watermark information. The parameters of the experiments are listed in Table 1.

In the experiments, the attacking strength means that the robustness index will be calculated on each interval of *S* (S∈[0,1]). In addition, the threshold of *BER* is set to 0.2 empirically, means that when *BER* < 0.2 the extracted watermark is regarded as the same as the original. For each interval of *S*, watermarking algorithms are executed 1000 times on each image with the cropping area randomly chosen. Finally, the robustness results from watermarking algorithms are compared with the theoretical ones, which are calculated by the proposed model.

#### 3.2.1. Experimental Results

Parts of the robustness results are given in Table 2, Table 3 and Table 4.

**Figure 11:**
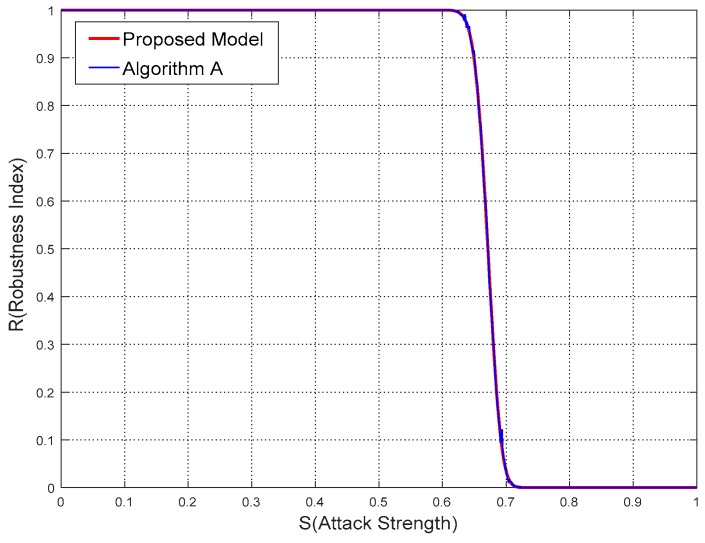
Comparisons of results from proposed model and Algorithm A in Experiment 1.

**Figure 12:**
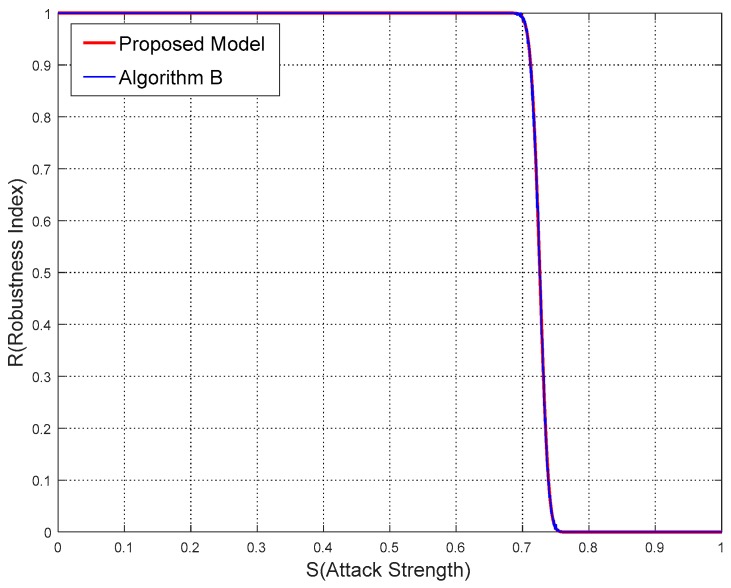
Comparisons of results from the proposed model and Algorithm B in Experiment 2.

**Figure 13:**
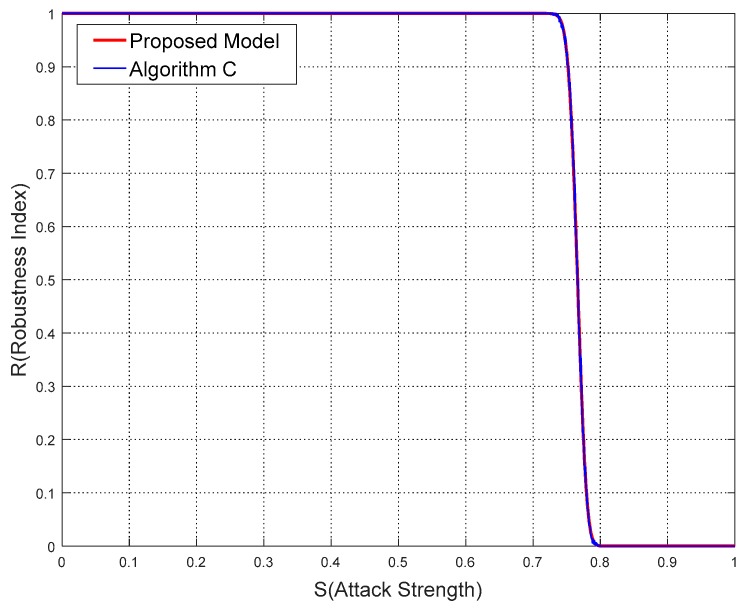
Comparisons of results from the proposed model and Algorithm C in Experiment 3.

From Table 2, Table 3 and Table 4, in terms of different parameters the robustness index *R* calculated by the proposed model and watermarking algorithms are obviously close to each other. More specifically, the comparisons of results between the proposed model and algorithms are drawn in Figure 11, Figure 12 and Figure 13.

From Figure 11, Figure 12 and Figure 13, it is not difficult to deduce that the robustness results calculated by the proposed model agree with the experimental ones. The curve shapes of the proposed model almost overlie those of the watermarking algorithms in these figures. While the sections of *R* starting to leave the value 1 and approach the value 0 are also nearly the same.

#### 3.2.2. Statistics Results

**Figure 14:**
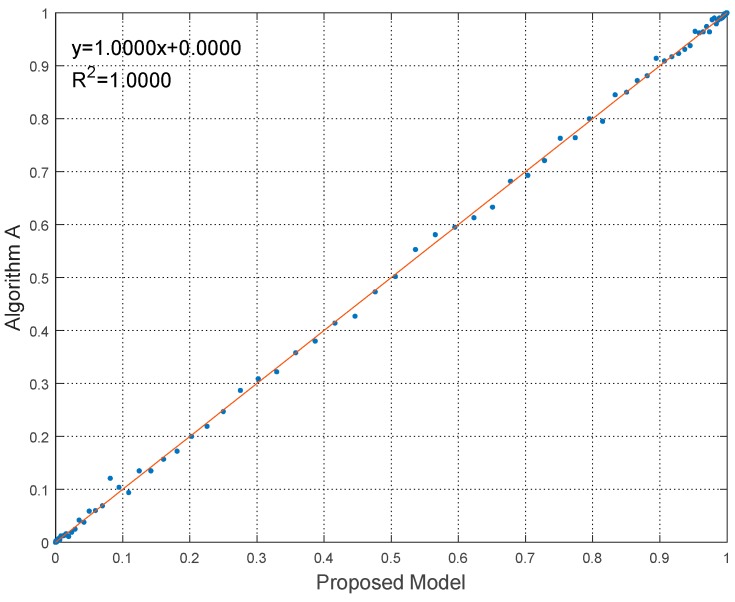
Linear regression of robustness results in Experiment 1.

**Figure 15:**
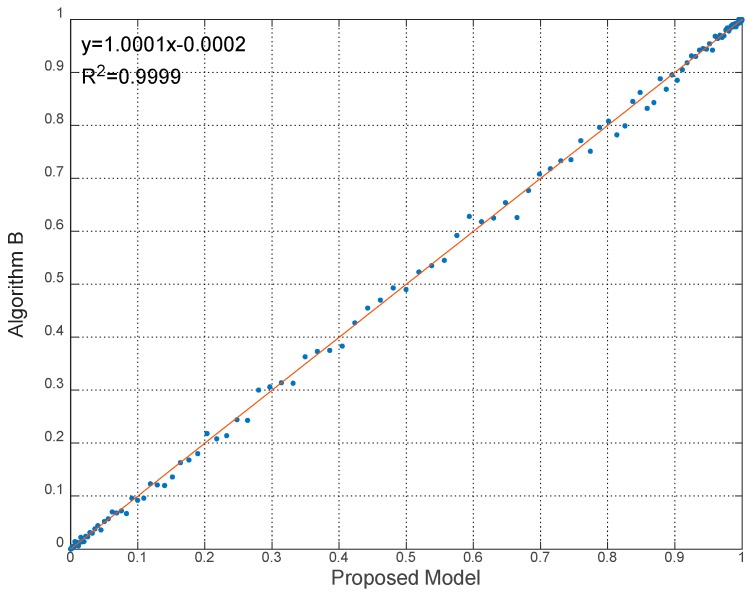
Linear regression of robustness results in Experiment 2.

**Figure 16:**
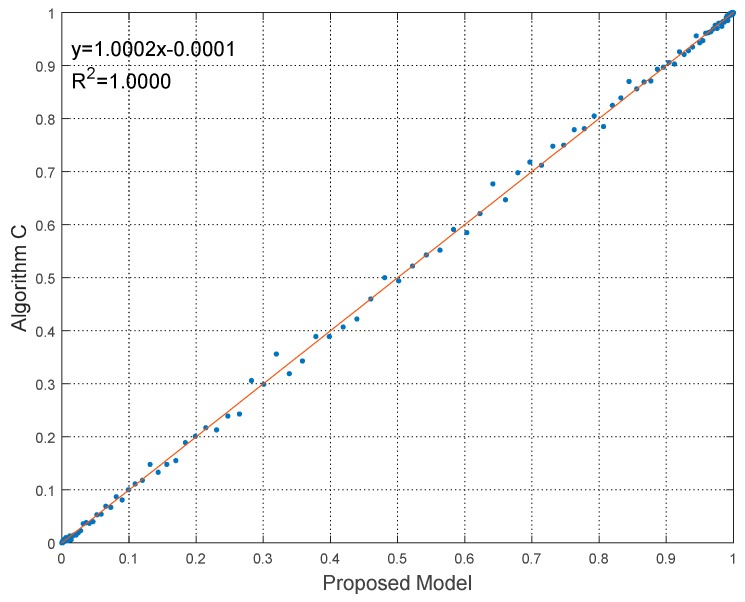
Linear regression of robustness results in Experiment 3.

To give the quantitative comparison results, the linear regressions of the experiments with the coefficient of determination ($R^{2}$) are shown in Figure 14, Figure 15 and Figure 16.

It can be seen from Figure 14, Figure 15 and Figure 16 that the linear relationships of robustness results calculated by proposed model and algorithms are evident and obvious, as the regression equations approaching y=x very closely. Besides, $R^{2}$ of the linear regressions is nearly equal to 1. Hence the linear relationship demonstrates the effectiveness to predict robustness results by using the proposed model. The statistical error results are also listed in Table 5. The statistics indices include the maximum value, the mean value and the standard deviation (Std) of errors.

From Table 5, it is reasonable to conclude that errors between the proposed model and watermarking algorithms are small enough to be ignored. The maximum errors do not exceed 0.05, and the mean values of errors are no more than 0.0002, with standard deviations less than 0.005, indicating that the errors have kept a low and stable level.

From the perspective of probability, the robustness result from a single procedure of watermark embedding and extraction can be regarded as a result of the Bernoulli Process. With the number of experiments increasing, the empirical robustness results will get closer to the theoretical ones, which are the expected probability of watermark has been successfully extracted. Thus, the experiments conducted above belong to the Monte Carlo method for estimating the theoretical robustness values. By using the Chernoff bound [43], if Pr represents the probability, *m* represents the number of experiments, *R* means the theoretical value, *R’* represents the empirical result, ε represents mean error ratio and δ represents the probability of false positive, the approximation for *R* satisfies:

(11) $$\mathrm{Pr}(\left|R^{\prime }-R\right|\geq \varepsilon R)\leq \delta ,$$

when:

(12) $$m\geq \frac{3\ln\frac{2}{\delta }}{\varepsilon^{2}R}.$$

Hence, the condition on *m* provides an (ε,δ)-approximation for *R*. Noted that *m* varies by different robustness result *R*. Taking *S =* 0.6700 and *R =* 0.5360 in Experiment 1 as an example, according to Table 2, if the approximation need to satisfy ε=(0.5530−0.5360)/0.5360=0.0317 and δ is set to 0.05 empirically, then the bound of *m* should be:

(13) m≥9279

by Equation (12). Apparently, the actual number of experiments in Experiment 1, which is 1000, is much smaller than the probabilistic bound of Equation (13). This situation demonstrates that the robustness results of the proposed method are close to empirical ones even of fewer experiments.

According to the statistic results and the probabilistic bound analyzed above, it is easy to deduce that the proposed robustness computation model is able to reveal the watermarking algorithmic robustness against image cropping effectively and accurately.

#### 3.2.3. Efficiency Results

The efficiency of the proposed model and watermarking algorithms are listed in Table 6.

The high-efficiency of the proposed model is deduced from two perspectives, which are the time and the processed data volume. The time cost by the proposed model is much less than those of watermarking algorithms, although the watermarking algorithms have efficient performance in watermark embedding and extraction. Furthermore, unlike the algorithms used in experiments, the proposed model is running based on a few lines of code without any sample data. Hence the volume of data processed by the proposed model is tiny compared with watermarking algorithms. These advantages in time and data volume, offered by the proposed model, will definitely promote the robustness evaluation and computation in watermarking algorithms, especially for huge data volume of remote sensing images in the big data era.

### 3.3. Deductions from Proposed Model

In watermarking algorithm research, the parameters of watermark information length *L* and repeat times *N* are important parameters. For those algorithms which conform to the proposed watermarking principles, regarding finding how to set or optimize these parameters to enhance the robustness needs to be solved. Based on the proposed model, the relationships among robustness index *R*, watermark information length *L* and repeat times *N* are deduced theoretically. Hence, the parameters of *L* and *N* can be adjusted in relation to the design or development of a watermarking algorithm, the aim of which is to enhance the robustness. This optimization is also, conducted without experiments. Detailed relationships are deduced as follows.

#### 3.3.1. The Relationship between Robustness *R* and Repeat Times *N*

**Figure 17:**
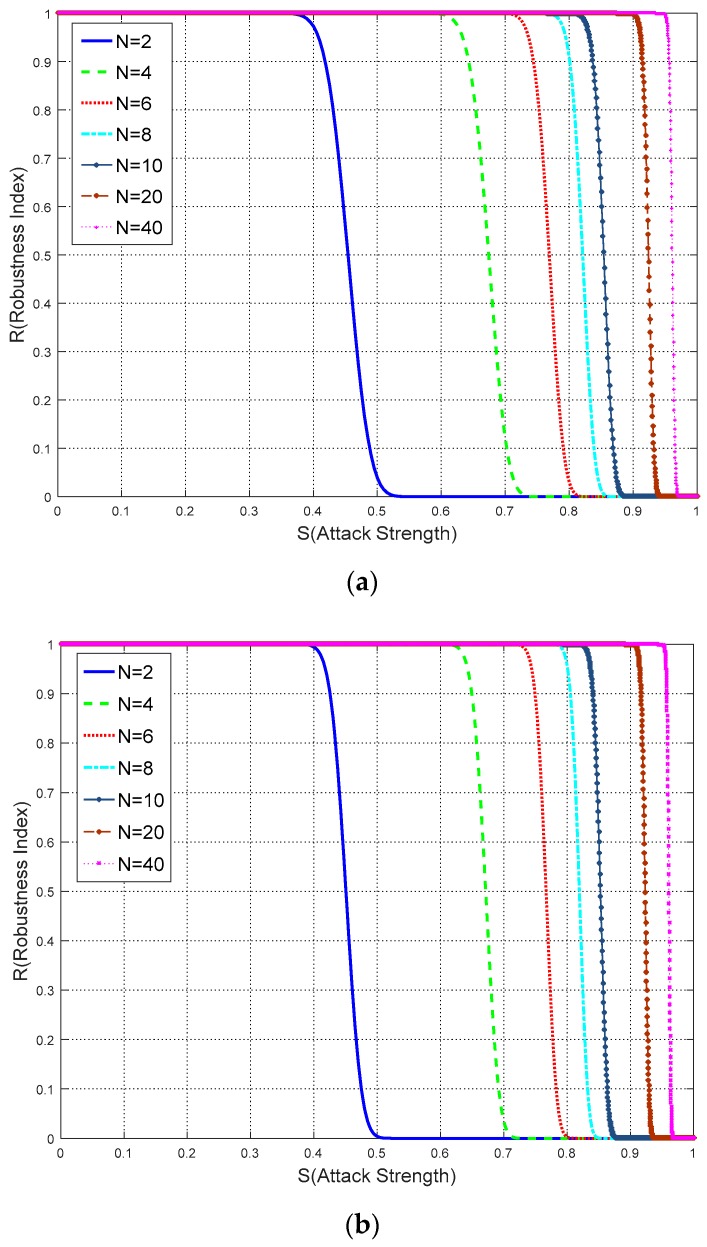

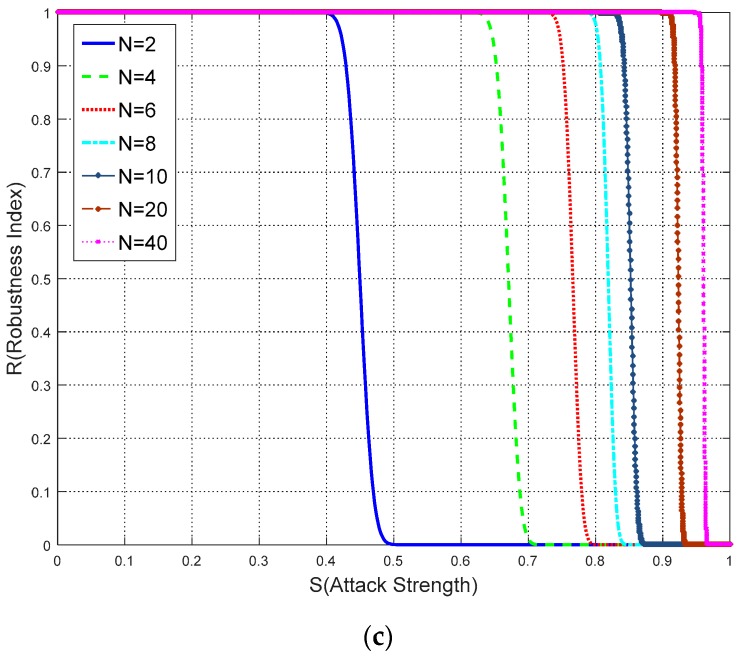
The relationship between robustness and watermark repeat times: (**a**) *L* = 100; (**b**) *L* = 200; (**c**) *L* = 300.

Intuitively, it may be felt that the more times watermark is embedded, the more likely the watermarking algorithmic robustness get enhanced. The computation experiment based on the proposed model has been conducted to verify this intuition. In the experiment, the binary watermark information length *L* is set to 100, 200, 300 and *N* is set to 2, 4, 6, 8, 10, 20, and 40 respectively. Then robustness index is calculated by the proposed model. Results of the relationship are shown in Figure 17.

As shown in Figure 17, two new results have been achieved as follows: with an increase in the watermark repeat times *N* and fixed *L*: (1) the image cropping robustness is enhanced; and (2) the rate of robustness improvement becomes slower. As shown by the curves of each *N* value, in those particular sections where *R* begins to drop, a larger *N* appears on the right side of those curves with a smaller *N*. For example: for each figure the curve in which *N =* 20 begins to drop near *S =* 0.9, while the curve in which *N* = 4 is near the line *S =* 0.6. Thus this phenomenon indicates that the robustness improvement is in accordance with the repeat of *N*. Additionally, the changed rate of the curves reveals a further interesting result. It can be deduced that when *N* becomes sufficiently large, the curves will then be reliably and sufficiently close to *R* = 1 and *S* = 1.

Based on the above results, new knowledge has been gained. Increasing watermark repeat times will definitely enhance the watermark robustness against image cropping attacks, however, embedding too much watermark information will cause significant distortions in remote sensing images. Hence, it is easily concluded that the robustness against image cropping can be enhanced by increasing the watermark repeat times to an appropriate value, or in other words, achieve the balance between robustness and imperceptibly.

#### 3.3.2. The Relationship between Robustness *R* and Watermark Information Length *L*

**Figure 18:**
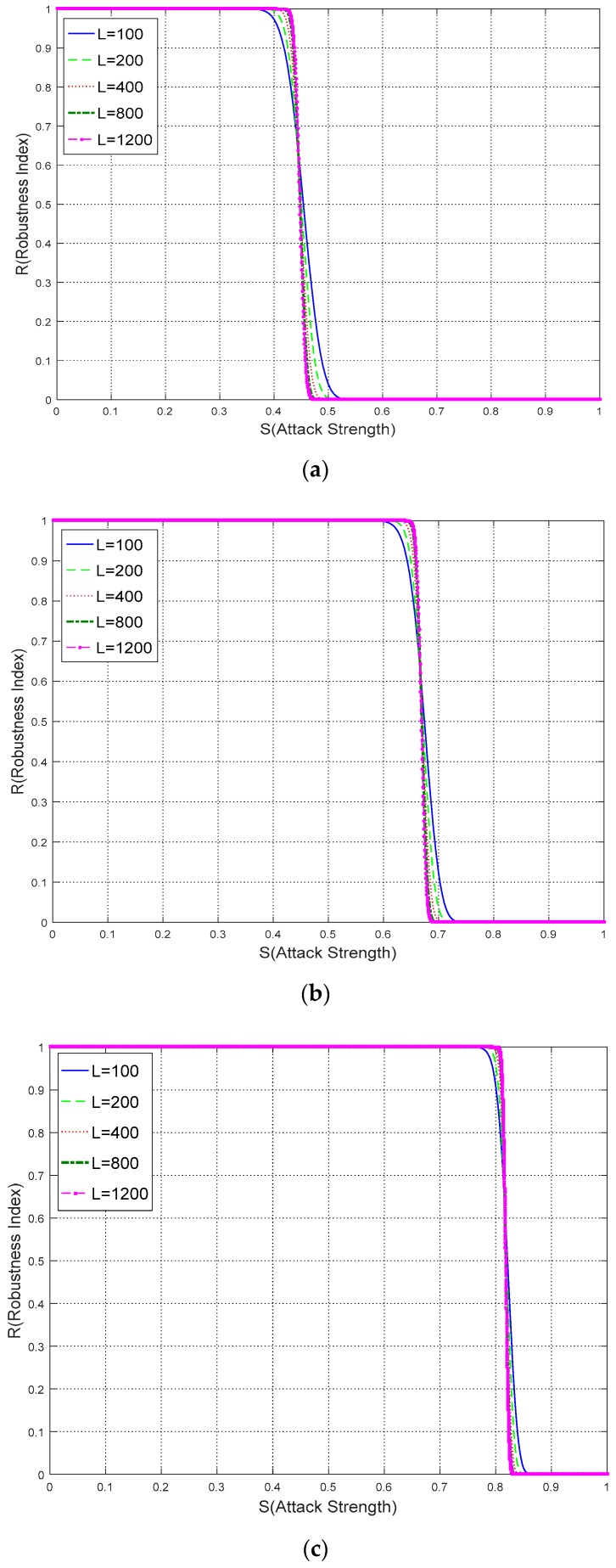
The relationship between robustness and watermark information length: (**a**) *N* = 2; (**b**) *N* = 4; (**c**) *N* = 8.

In this experiment, the relationship between robustness *R* and watermark information length *L* is explored. For watermarking algorithms, the watermark may vary between a binary sequence with a length of less than 200 or a binary image with its length of more than 1024. Hence it is of importance to determine whether there is a strong connection between robustness and watermark information length. Based on the proposed computation model, *N* is set as 2, 4, 8 and *L* is set to 100, 200, 400, 800, and 1200 respectively. Figure 18 shows the robustness comparisons with different *L*.

From Figure 18, although *L* varies from 100 to 1200 in the three figures, only minor differences exist where the curves approaching *R* = 1 and *R* = 0. While the trends of those curves and the sections, where *R* begins to drop, are similar and close. Consequently, it is reasonable to infer that the watermark robustness is obviously not determined by watermark information length.

According to the above result, two conclusions are deduced as follows: (1) Increasing *L* alone, evidently cannot improve the associated robustness. Considering the watermark capacity limitation, *L* is suggested not be an excessively large number, for example, 1000 for a pseudo-random binary sequence; (2) The robustness computation of a large *L* can be estimated instead of a much smaller *L*. For example, in the above experiments, the curve of *L* = 1200 is very close to the curve of *L* = 400. By using this conclusion, the computation complexity is further reduced dramatically when *L* becomes large, which provide an efficient method to estimate the watermark robustness.

## 4. Discussion

From the experimental results of the proposed model verification, it can be seen that as long as the watermarking algorithm is consistent with the fundamental principles, the algorithmic robustness against image cropping attack can be determined by the proposed model theoretically. According to the properties of the proposed model, the calculation process has no relationship to experimental data, and avoids the computational resource cost of experiments. Hence, it provides a theoretical method to evaluate the robustness against image cropping attack. In specific applications of watermarking remote sensing images, the model helps the copyright owner consider how much of the image may be cropped and decide the parameters of watermarking algorithms. Especially for the massive data providers, the theoretical calculation saves much energy and cost compared to practical experiments.

Further applicability improvements have also been made by the deduction experiments based on the proposed model. The relationships among robustness index, watermark information length and watermark repeat times have been investigated and revealed by these experiments. Thus, these enlightenments can be used to facilitate the design of watermarking algorithms which obey the proposed watermarking principles. For example, considering the fidelity requirement of remote sensing images, the total embedded watermark bits, which equals *L × N*, are strictly restricted. To increase the robustness against image cropping for existing watermarking algorithms, it is significant to increase *N* and decrease *L* without causing prominent distortions at the same time. Another example may be when watermarking remote sensing images in the form of big data. In this situation, larger *L*, which is necessary for copyright identification, will increase the complexity and the time of robustness computation. From the experimental results, smaller *L* can be used to reduce the computation time and provide the efficient method to accurately estimate the robustness.

Another potential application of the proposed model may be the promotion on research on robustness computation models against other kinds of attacks. The analysis of other image attacks needs further and detailed study, hence some views discussed here are incomplete. Generally, image attacks can be divided into two categories: content/pixel attacks and geometric transformation attacks. For example, the attacks of contents include filtering, JPEG compression (a standard created by the Joint Photographic Experts Group), noise addition and so on, while the attacks of geometric transformation include rotation, scaling, other affine transformations and so on. We note that the process of geometric attacks, as the compound of image geometric transformation and image contents resampling, seem to be more complicated in robustness analysis comparing with attacks purely on contents. Among the attacks mentioned above, the analysis principles of image cropping attack are simpler as watermark synchronization of the remaining part keep correct. Further study may be conducted on the basis of principles proposed in this paper and combined with the characteristics of various attacks.

## 5. Conclusions

The objective of this study, which is to theoretically calculate the watermarking algorithmic robustness against image cropping attack for remote sensing images, has been realized. The proposed model comprises analysis principles deduced from well-designed watermarking algorithms, probability-based evaluation index and robustness computation method. The robustness results calculated by the proposed model have been compared to experimental ones, and the fact that errors are very small, demonstrate the effectiveness and accuracy of the proposed model. Furthermore, based on the proposed model, the relationships of the robustness, watermark information length and watermark repeat times have been deduced as long as the watermarking algorithms conforming to the proposed principles. The deductions can be used to optimize the watermarking algorithm parameters and enhance the robustness against image cropping. In summary, the proposed robustness computation model provides an effective method to analyze and calculate the watermarking algorithmic robustness, and it can be adopted to facilitate the improvement of watermark robustness. Further work will attempt to analyze other types of attacks on remote sensing images and extend the applicability of robustness computation model for them.

## Figures and Tables

**Table 1:** Experimental parameters (*L* = 200 and *N* = 4).

Experiments	Watermark Length (*L*)	Watermark Repeat Time (*N*)	Attacking Strength Interval	Comparison Algorithm
Experiment 1	200	4	1/800	Algorithm A [11]
Experiment 2	400	5	1/2000	Algorithm B [8]
Experiment 3	300	6	1/1800	Algorithm C [10]

**Table 2:** Part of robustness results in Experiment 1.

*S* (Attack Strength)	*R* (Robustness Index)		*S* (Attack Strength)	*R* (Robustness Index)	
	Proposed Model	Algorithm A		Proposed Model	Algorithm A
0.2000	1	1	0.6700	0.5360	0.5530
0.5000	1	1	0.6800	0.3018	0.3090
0.6000	1	1	0.6900	0.1247	0.1350
0.6100	0.9996	1	0.7000	0.0351	0.0420
0.6200	0.9980	0.9970	0.7100	0.0100	0.0050
0.6300	0.9905	0.9890	0.7200	0.0006	0.0010
0.6400	0.9645	0.9640	0.7300	0	0
0.6500	0.8945	0.9140	0.7400	0	0
0.6600	0.7516	0.7630	0.7500	0	0

**Table 3:** Part of robustness results in Experiment 2.

*S* (Attack Strength)	*R* (Robustness Index)		*S* (Attack Strength)	*R* (Robustness Index)	
	Proposed Model	Algorithm A		Proposed Model	Algorithm A
0.2000	1	1	0.7300	0.3493	0.3630
0.5000	1	1	0.7350	0.1895	0.1800
0.6800	1	1	0.7400	0.0832	0.0670
0.6900	0.9994	1	0.7450	0.0287	0.0310
0.7000	0.9910	0.9860	0.7500	0.0076	0.0070
0.7100	0.9309	0.9300	0.7550	0.0015	0.0010
0.7150	0.8483	0.8620	0.7600	0.0002	0
0.7200	0.7145	0.7180	0.7700	0	0
0.7250	0.5378	0.5350	0.7800	0	0

**Table 4:** Part of robustness results in Experiment 3.

*S* (Attack Strength)	*R* (Robustness Index)		*S* (Attack Strength)	*R* (Robustness Index)	
	Proposed Model	Algorithm A		Proposed Model	Algorithm A
0.2000	1	1	0.7650	0.5427	0.5430
0.5000	1	1	0.7700	0.3583	0.3430
0.7300	0.9990	0.9980	0.7750	0.1987	0.2010
0.7350	0.9966	0.9960	0.7800	0.0898	0.0810
0.7400	0.9892	0.9900	0.7850	0.0319	0.0360
0.7450	0.9698	0.9690	0.7900	0.0087	0.0060
0.7500	0.9265	0.9210	0.7950	0.0017	0.0020
0.7550	0.8445	0.8390	0.8	0.0002	0
0.7600	0.7142	0.7120	0.805	0	0

**Table 5:** Error statistics of robustness results between proposed model and algorithms.

Experiments	Max	Mean	Std
Experiment 1	0.0396	0.0002	0.0028
Experiment 2	0.0390	−0.0001	0.0027
Experiment 3	0.0365	−0.0001	0.0025

**Table 6:** Computation time and processed data volume cost by the proposed model and algorithms.

Experiments	Proposed Model		Watermarking Algorithms	
	Time	Data Volume	Time	Data Volume
Experiment 1	1175 s	<1 MB	20,576 s	≈601,000 MB
Experiment 2	3298 s	<1 MB	67,510 s	≈601,000 MB
Experiment 3	1790 s	<1 MB	24,792 s	≈601,000 MB

## Appendix A. MATLAB Code of the Proposed Robustness Computation Model

Code file name: OPDrawDeleteRobustness.m
Contents:
function [DeleteRate, Prob] = OPDrawDeleteRobustness(WatermarkLength, RepeatTimes,
NCThreshold, DeleteStartRate, DeleteEndRate, DeleteIntervalRate)
     tic;
     TotalWMBits = WatermarkLength × RepeatTimes;
     DeleteRate = DeleteStartRate:DeleteIntervalRate:DeleteEndRate;
     Count = (DeleteEndRate − DeleteStartRate)/DeleteIntervalRate + 1;
     Count = round(Count);
     Prob = ones(1, Count);
     parfor i = 1:Count    %parallel computing to improve the efficiency
          TempRate = DeleteRate(i);
          DeleteBits = round(TotalWMBits × TempRate);
          CurR = OPCalDetectSucceesPro(WatermarkLength, RepeatTimes, DeleteBits, 
          NCThreshold);
          Prob(i) = CurR;
     end
     plot(DeleteRate, Prob);
     toc;
end
 
Code file name: OPCalDetectSuccessPro.m
Contents:
function DetectSuccessPro = OPCalDetectSucceesPro(WatermarkLength, RepeatTimes, DeleteBits, 
NCThreshold)
     L = WatermarkLength;
     N = RepeatTimes;
     D = DeleteBits;
     X = ceil(L × NCThreshold);
     if D > = L × N
          DetectSuccessPro = 0;
     elseif D < N
          DetectSuccessPro = 1;
     else
          for i = X:L
              tempPro = OPCalMPProbDirectly(i, N, D – N × (L − i), L, N, D);
              Sum = Sum + tempPro;
          end
          DetectSuccessPro = Sum;
          if DetectSuccessPro < 0
              DetectSuccessPro = 0;
          end
          if DetectSuccessPro > 1
              DetectSuccessPro = 1;
          end
     end
end
 
Code file name: OPCalMPProbDirectly.m
Code:
function Result = OPCalMPProbDirectly(dL, dN, dD, dtotalL, dtotalN, dtotalD)
     %mp is the data type of Multiprecision Computing Toolbox for MATLAB (detailed
     information in www.advanpix.com), which is used to calculate the probability with
     sufficient precision.
     L = mp(dL);
     N = mp(dN);
     D = mp(dD);
     totalL = mp(dtotalL);
     totalN = mp(dtotalN);
     totalD = mp(dtotalD);
     totalbits = totalL × totalN;
     if D < 0
          Result = 0;
     elseif D = = 0
          Result = exp(gammaln(totalL + 1) − gammaln(L + 1) − gammaln(totalL − L + 1) − (gammaln(totalbits + 1) − gammaln(totalD + 1) − gammaln(totalbits − totalD + 1)));
     elseif D < N
          Result = exp(gammaln(totalL + 1) − gammaln(L + 1) − gammaln(totalL − L + 1) + gammaln(L × N + 1) − gammaln(D + 1) − gammaln(L × N − D + 1) − (gammaln(totalbits + 1) − gammaln(totalD + 1) − gammaln(totalbits − totalD + 1)));
     elseif D > L × (N − 1)
          Result = 0;
     else
          MaxCrackColCount = fix(D/N);
          sum = exp(gammaln(totalL + 1) − gammaln(L + 1) − gammaln(totalL − L + 1) + gammaln(L × N + 1) − gammaln(D + 1) − gammaln(L × N − D + 1) − (gammaln(totalbits + 1) − gammaln(totalD + 1) − gammaln(totalbits − totalD + 1)));
          for i = 1:MaxCrackColCount
              temp = (−1)^i × exp(gammaln(totalL + 1) − gammaln(L + 1) − gammaln(totalL − L + 1) + gammaln(L + 1) − gammaln(i + 1) − gammaln(L − i + 1) + gammaln((L − i) × N + 1) − gammaln(D − N × i + 1) − gammaln((L − i) × N − D + N × i + 1) − (gammaln(totalbits + 1) − gammaln(totalD + 1) − gammaln(totalbits − totalD + 1)));
              sum = sum + temp;
          end
          Result = sum;
     end
end
